# COVID-19 incidence among nonphysician healthcare workers at a tertiary care center–Iowa, 2020–2021

**DOI:** 10.1017/ash.2022.65

**Published:** 2022-05-16

**Authors:** Takaaki Kobayashi, John Heinemann, Alexandra Trannel, Alexandre Marra, William Etienne, Oluchi Abosi, Stephanie Holley, Mary Kukla, Angie Dains, Kyle Jenn, Holly Meacham, Beth Hanna, Bradley Ford, Melanie Wellington, Patrick Hartley, Daniel Diekema, Jorge Salinas

## Abstract

**Background:** Whether working on COVID-19 designated units put healthcare workers (HCWs) at higher risk of acquiring COVID-19 is not fully understood. We report trends of COVID-19 incidence among nonphysician HCWs and the association between the risk of acquiring COVID-19 and work location in the hospital. **Methods:** The University of Iowa Hospitals & Clinics (UIHC) is an 811-bed, academic medical center serving as a referral center for Iowa. We retrospectively collected COVID-19–associated data for nonphysician HCWs from Employee Health Clinic between June 1st 2020 and July 31th 2021. The data we abstracted included age, sex, job title, working location, history of COVID-19, and date of positive COVID-19 test if they had a history of COVID-19. We excluded HCWs who did not have a designated working location and those who worked on multiple units during the same shift (eg, medicine resident, hospitalist, etc) to assess the association between COVID-19 infections and working location. Job titles were divided into the following 5 categories: (1) nurse, (2) medical assistant (MA), (3) technician, (4) clerk, and (5) others (eg patient access, billing office, etc). Working locations were divided into the following 6 categories: (1) emergency department (ED), (2) COVID-19 unit, (3) non–COVID-19 unit, (4) Clinic, (5) perioperative units, and (6) remote work. **Results:** We identified 6,971 HCWs with work locations recorded. During the study period, 758 HCWs (10.8%) reported being diagnosed with COVID-19. Of these 758 COVID-19 cases, 658 (86.8%) were diagnosed before vaccines became available. The location with the highest COVID-19 incidence was the ED (17%), followed by both COVID-19 and non–COVID-19 units (12.7%), clinics (11.0%), perioperative units (9.4%) and remote work stations (6.6%, p **Conclusions:** Strict and special infection control strategies may be needed for HCWs in the ED, especially where vaccine uptake is low. The administrative control of HCWs working remotely may be associated with a lower incidence of COVID-19. Given that the difference in COVID-19 incidence among HCWs by location was lower and comparable after the availability of COVID-19 vaccines, facilities should make COVID-19 vaccination mandatory as a condition of employment for all HCWs, especially in areas where the COVID-19 incidence is high.

**Funding:** None

**Disclosures:** None

